# Recent progress of electrochemical hydrogen evolution over 1T-MoS_2_ catalysts

**DOI:** 10.3389/fchem.2022.1000406

**Published:** 2022-10-07

**Authors:** Yicen Zhang, Li Wang, Qian Chen, Jing Cao, Cen Zhang

**Affiliations:** Department of Chemistry and Chemical Engineering, Hunan Institute of Science and Technology, Yueyang, Hunan, China

**Keywords:** hydrogen evolution reaction, 1T-MoS_2_, phase modulation, characterization, electrocatalysts

## Abstract

Developing efficient and stable non-noble metal catalysts for the electrocatalytic hydrogen evolution reaction (HER) is of great significance. MoS_2_ has become a promising alternative to replace Pt-based electrocatalysts due to its unique layered structure and adjustable electronic property. However, most of the reported 2H-MoS_2_ materials are stable, but the catalytic activity is not very ideal. Therefore, a series of strategies such as phase modulation, element doping, defect engineering, and composite modification have been developed to improve the catalytic performance of MoS_2_ in the HER. Among them, phase engineering of 2H-MoS_2_ to 1T-MoS_2_ is considered to be the most effective strategy for regulating electronic properties and increasing active sites. Hence, in this mini-review, the common phase modulation strategies, characterization methods, and application of 1T-MoS_2_ in the HER were systematically summarized. In addition, some challenges and future directions are also proposed for the design of efficient and stable 1T-MoS_2_ HER catalysts. We hope this mini-review will be helpful to researchers currently working in or about to enter the field.

## Introduction

Hydrogen (H_2_) is considered a promising renewable energy source because of high energy density and zero pollution (Lee et al., 2021; Zhang L. et al., 2022). Nowadays, H_2_ is produced on an industrial scale through methane reforming or coal gasification, which inevitably releases a large amount of greenhouse gases (Li et al., 2022a; Li et al., 2022b; Yang et al., 2022). By contrast, the electrochemical hydrogen evolution reaction (HER) from water-splitting is more environmentally friendly because the electricity could be derived from solar energy or wind power (Xiang et al., 2021; Ye et al., 2021; Gong et al., 2022). As the best catalyst for the HER, the high price and low reserves of platinum (Pt) make it unable to meet the needs of industrialization. Hence, developing non-noble metals with abundant reserves and low prices to efficiently catalyze the HER is still challenging.

**FIGURE 1:**
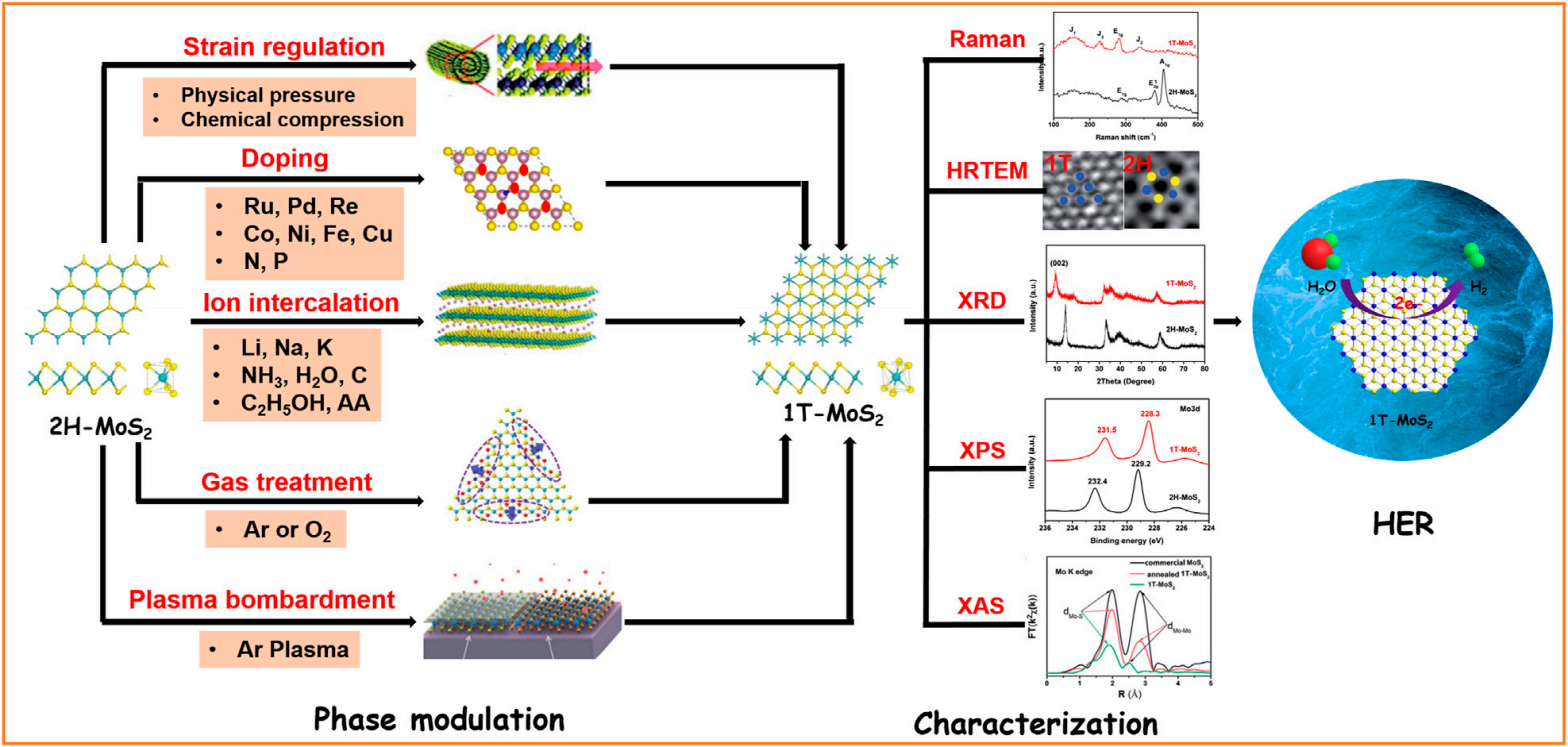
Schematic summary of phase modulation, characterization techniques, and HER application of 1T-MoS_2_.

As a typical two-dimensional material, MoS_2_ showed great potential to replace Pt theoretically and experimentally (Cao et al., 2021b; Gong et al., 2021; Li X.-Y. et al., 2022). The monolayer of MoS_2_ is connected by the S–Mo–S covalent bond, where different arrangements of Mo and S layers will result in the formation of different crystal phases, such as 1T, 2H, and 3R. For catalysis, 2H-MoS_2_ and 1T-MoS_2_ are most used and compared (Gong et al., 2020; Li et al., 2021; Zhang et al., 2022a). The 2H phase possesses triangular prism coordination with semiconducting properties and is thermodynamically stable. However, the 1T phase possesses octahedral coordination and is a metastable phase with metallic properties (Tang and Jiang, 2015). Due to the different crystal structures, the physicochemical properties of 1T and 2H phases show great differences. The electronic conductivity of metallic 1T-MoS_2_ is about five orders of magnitude higher than that of 2H-MoS_2_. In addition, it has active centers on both the basal and edge planes, while 2H-MoS_2_ only exhibits catalytic activity on the edge planes (Tang and Jiang, 2016). In the past decades, most of the reported MoS_2_ electrocatalysts are 2H-MoS_2_ due to the thermodynamic instability of the 1T phase. Hence, phase modulation from 2H to 1T has been achieved by lateral translation of the S plane and changing the filling state of Mo 3d orbitals. Although a series of regulation strategies have been developed in recent years, there are few systematic reviews on the targeted synthesis and HER application of 1T-MoS_2_. Therefore, we have summarized some common preparation methods, necessary characterization techniques of 1T-MoS_2_, and its application in the HER (Figure 1). Finally, the challenges for targeted synthesis and rational design of advanced 1T-MoS_2_ electrocatalysts are also proposed.

### Characterization techniques for 1T-MoS_2_

Usually, the 1T phase and 2H phase coexist in the as-synthesized MoS_2_ materials. Therefore, it is necessary to analyze the 1T phase qualitatively and quantitatively, which plays an important role for studying the structure–performance relationship and developing high-performance MoS_2_-based electrocatalysts. Due to the huge structure difference between 1T-MoS_2_ and 2H-MoS_2_, the characterization of 1T-MoS_2_ could be conducted by X-ray diffraction (XRD), Raman spectroscopy (Raman), X-ray photoelectron spectroscopy (XPS), high-resolution transmission electron microscopy (HRTEM), and X-ray absorption spectroscopy (XAS).

XRD is frequently utilized to reflect the lattice parameter changes caused by the phase transition from 2H-MoS_2_ to 1T-MoS_2_. 1T-MoS_2_ obtained by phase transition through guest molecule intercalation will increase the interlayer spacing, which is accompanied by a peak downshift at 2θ = 14^o^, corresponding to the (002) crystal planes of 2H-MoS_2_. According to the literature reports, the diffraction peak of 1T-MoS_2_ obtained by NH_3_ (Liu et al., 2015; Zhang et al., 2022a) and Na^+^(Wang X. et al., 2014) intercalation shifted from 14^o^ to 9.4^o^ and 12.4^o^, respectively. This result indicates that the magnitude of the peak shift depends on the size of intercalated molecules or ions. However, it is difficult to observe the characteristic diffraction peak of single- or few-layer 1T-MoS_2_ obtained by exfoliation due to the weak crystallinity (Tong et al., 2017). In addition, only the diffraction peaks of 2H-MoS_2_ could be detected when the 1T phase content in 2H/1T-MoS_2_ is low, and the existence of the 1T phase needs to be further determined by Raman or XPS (Cao et al., 2022).

Raman spectroscopy is a simple and effective technique to determine the existence of 1T-MoS_2_. Typically, 2H-MoS_2_ shows two characteristic bands of E^1^_2g_ and A_1g_ at 383 and 408 cm^−1^, respectively (Zhang et al., 2021). However, a set of new bands at 152, 226, and 330 cm^−1^ appeared with the presence of the 1T phase (Liu Z. et al., 2017). In addition, it is difficult to observe these characteristic peaks sometimes with the low proportion of the 1T phase in the 1T/2H-MoS_2_ composite, which requires further verification by XPS (Pradhan and Sharma, 2019). Meanwhile, it should be emphasized that the laser power of the Raman test is usually less than 0.1 mW because high laser power could destroy the structure of MoS_2_ and even burn the sample.

XPS is the only characterization method which could quantitatively analyze the content of the 1T phase. It has been the preferred analysis method for researchers because of its simple operation. According to previous studies, MoS_2_ exists as a 2H phase with semiconductor properties when the Mo 3d orbitals are fully filled. When the electrons are partially filled, MoS_2_ is a 1T phase with metallic properties (Voiry et al., 2015). Generally, the binding energies of Mo^4+^ 3d_5/2_ and Mo^4+^ 3d_3/2_ in 1T-MoS_2_ are 231.5 and 228.3 eV, respectively, which are 0.9 eV lower than those of 2H-MoS_2_ (232.4 and 229.2 eV) due to fewer electrons being filled (Wang et al., 2017; Cao et al., 2021a; Zhang et al., 2022b). Additionally, the 1T/2H phase proportion could be easily obtained by peak fitting in the Mo 3d region.

The atomic arrangement of MoS_2_ could be directly observed by HRTEM to distinguish the crystal phase. As a layered compound, the structure difference between 1T-MoS_2_ and 2H-MoS_2_ can be visualized from the top view ([001] plane) and the side view ([100] plane). From the enlarged image on the basal plane, 1T-MoS_2_ displays a typical triangular configuration with octahedral coordination, while 2H-MoS_2_ exhibits a honeycomb configuration with trigonal prism coordination (Sun et al., 2018). By observing from the edge plane, the S–Mo–S coordination of 1T-MoS_2_ shows a chevron configuration, while 2H-MoS_2_ exhibits a diagonal line pattern (Enyashin et al., 2011). Since it is difficult for operators to select a side view, most literature works adopt the atomic structure of the basal plane to confirm the existence of the 1T phase.

XAS is a newly developed technique for analyzing the structure and phase of MoS_2_. The phase change between 1T and 2H is detected by observing the signal vibration caused by scattering of incident photoelectrons between two surrounding atoms. Furthermore, the bond length of MoS_2_ could be determined by Fourier transform of the obtained Mo K-edge spectra (Yang et al., 2017). Deng et al. found that the bond length and peak strength of Mo–Mo and Mo–S bonds in 1T-MoS_2_ were smaller than those in 2H-MoS_2_ (Deng et al., 2019). However, characterization and data analysis of XAS require a high cost and rich experience, which makes it a limited technique utilized by researchers.

### Phase modulation strategies of 1T-MoS_2_

Generally, 1T-MoS_2_ does not exist in nature because of its metastable property. At present, the synthesis strategy in the laboratory is to convert 2H-MoS_2_ into 1T-MoS_2_ through phase modulation. So far, some strategies such as ion intercalation, doping, strain regulation, gas treatment, and plasma bombardment have been reported to realize the targeted phase transition successfully.

Since the adjacent layers of MoS_2_ were connected by the weak van der Waals force so that alkali metals (Li, Na, and K), small inorganic molecules (NH_3_, H_2_O (Geng et al., 2017), and RGO (Mahmood et al., 2016)), and organic molecules (alcohol or organic acid) could be inserted into the interlayers easily. During the intercalation process, the electrons of guest species are transferred to the Mo3d orbitals to change the filling state, which results in partial conversion of the originally stable 2H phase into the 1T phase. In addition, the intercalated molecules will be positively charged. Chemical lithium intercalation is the most common and mature intercalation method to obtain 1T-MoS_2_ (Lukowski et al., 2013; Tan et al., 2018; Xu et al., 2019). Typically, bulk MoS_2_ powders are immersed into an excess n-butyllithium solution for 6–72 h at room temperature in a glove box. With the assistance of ultrasonication, single- or few-layer 1T-MoS_2_ nanosheets are obtained. The 1T phase content in the exfoliated MoS_2_ products is affected by lithium time, solvent, and temperature, and a highest content of the 70% 1T phase could be obtained (Zheng et al., 2014). In a similar procedure, Na^+^(Gao et al., 2015) and K^+^(Zhang et al., 2016) could also be intercalated into the interlayer space, thus inducing 2H to 1T phase transition. In order to further increase the 1T content, a liquid-ammonia-assisted lithiation (LAAL) method was developed to greatly enhance the intensity of the lithium process, resulting in monolayer porous MoS_2_ nanosheets with a 1T content of about 81% (Yin et al., 2016). Considering the disadvantages such as high risk and uncontrollable insertion degree for alkali metal chemical intercalation, electrochemical intercalation has been extensively explored recently. The biggest difference between these two methods is that the driving force of electrochemical intercalation is much larger, thus exhibiting better controllability and higher efficiency (Wang X. et al., 2014; Chen et al., 2018). For example, Chen et al. prepared monolayer MoS_2_ quantum dots with size of 3–5 nm and 1T phase content of 92–97% by a quasi-full electrochemical process, which was achieved by a greatly increased Li intercalation content (Chen et al., 2018).

Small inorganic molecules such as NH_3_ are also an appropriate intercalation candidate to trigger phase transition. Usually, if excessive precursors containing -NH_2_ such as thiourea (Zhang et al., 2022a), thioacetamide (Liu Q. et al., 2017), urea (Sun et al., 2018), or ammonium bicarbonate (Wang et al., 2017) are added in the hydrothermal/solvothermal synthesis of MoS_2_, some NH_3_ molecules generated by hydrolysis could be easily inserted into the interlayer to obtain 1T-MoS_2_. Meanwhile, it should be noted that the hydrothermal temperature needs to be lower than 200 °C because the 1T phase is unstable at high temperatures. As an example, Sun et al. synthesized MoS_2_ nanosheets with a 1T phase fraction of 16.4%–90.2% by introducing different amounts of urea in the hydrothermal system (Sun et al., 2018). Liu et al. prepared a 1T-MoS_2_/single-walled carbon nanotube by adding excess thioacetamide to provide intercalated NH_4_^+^, which obtained the 1T phase content of around 60% (Liu Q. et al., 2017). However, according to a recent report, metallic 1T-MoS_2_ obtained by NH_4_^+^ was unstable and changed back to 2H-MoS_2_ spontaneously after exposing in air for 15 days. Thus, the authors developed a two-step solvothermal strategy which adopted organic solvents such as methanol, ethanol, isopropanol, or butanol to treat NH_4_^+^-intercalated 1T-MoS_2_ again. Interestingly, the C_2_H_5_OH-intercalated 1T-MoS_2_ preserved the 1T structure well after being stored in air for 360 days, where the superior stability was attributed to the strong interaction between ethanol and the MoS_2_ surface (Li et al., 2020). In addition, ascorbic acid (AA) has also been reported as an excellent intercalation molecule to synthesize high stable 1T-MoS_2_ *via* a one-step hydrothermal method (Wang et al., 2022).

Doping heteroatoms into the lattice of 2H-MoS_2_ is considered an efficient way to modulate local electronic properties and induce 2H-to 1T transformation. The doped elements can be metals (Re, Co, Ru, Cu, Pd (Luo et al., 2018), Fe (Zhao et al., 2017), and Ni (Wang et al., 2022)) or non-metals (N and P), and it must be incorporated uniformly at the atomic scale. The doping technique can adopt a one-step method or post-treatment modification method. Considering Re has a close atomic radius of Mo and ReS_2_ has a similar structure as distorted 1T-MoS_2_, Xia et al. synthesized Re-doped 1T/2H-MoS_2_ nanosheets through a hydrothermal and anneal process (Xia et al., 2018). Ji et al. utilized a one-pot solvothermal strategy to prepare single-atom Cu-doped MoS_2_ nanoflowers with a large fraction of the 1T phase. In the solvothermal procedure, the Cu precursor reacted with MoS_3_ and donated electrons to MoS_2_, thus inducing and stabilizing the 1T phase (Ji et al., 2019). Similarly, Co-doped MoS_2_ nanosheets with a dominant metallic 1T phase were synthesized by doping Co^2+^ into the basal planes and S-edge planes (Nethravathi et al., 2017). Atomic Ru was incorporated into MoS_2_ nanosheets (SA-Ru-MoS_2_) using a simple one-step impregnation method, where Ru substituted lattice Mo atoms and triggered local phase transition (Zhang et al., 2019). Non-metal elements such as nitrogen or phosphorus were also frequently doped into MoS_2_ to modulate phase transition. Wang et al. embedded P into the lattice of 2H-MoS_2_ by a simple one-pot annealing method to induce partial phase transition and obtained ultra-stable in-plane 1T-2H/MoS_2_ heterostructures (Wang et al., 2018). Deng et al. prepared N-doped and PO_4_^3-^-intercalated MoS_2_ arrays by annealing with NaH_2_PO_4_ in NH_3_ atmosphere (Deng et al., 2019). Both the N doping and PO_4_^3-^ intercalation contributed to the formation of the 1T phase and acted synergistically, which induced about 41% of the 2H phase to 1T phase.

Recently, a strain regulation strategy has been developed to tune the electronic structure and realize the phase modulation of MoS_2_. Chi et al. reported that 2H to 1T phase transition occurred at an extremely high physical pressure (> 20 GPa) through layer sliding, which was evidenced by Raman spectra and XRD patterns (Chi et al., 2014). Similarly, chemical compressive force caused by bending also can result in gliding of the S plane and phase transition. Hwang et al. adopted N-(2-aminoethyl)-3α-hydroxy-5β-cholan-24-amide as a self-assembling material to roll up the exfoliated 2H-MoS_2_ nanosheets, which obtained 1T@2H nanoscrolls with a 1T phase content of 58% and high stability after heated at 200 C (Hwang et al., 2017).

Sulfur vacancies (Vs) produced by gas treatment or plasma bombardment could play the role of electron donors to trigger local phase transition. Yang et al. used Ar and O_2_ to treat monolayer MoS_2_, which leads to partial formation of the 1T phase by modulating defect configuration (Yang et al., 2016). Meanwhile, it should be pointed out that Vs were formed within the basal plane and at the edge planes by Ar and O_2_ treatment, respectively, and the 1T phase percentage was higher for O_2_-treated samples due to a stronger phase-driven force. Zhu et al. reported a facile and controllable Ar bombardment technique to produce single Vs and induce phase transition on monolayer 2H-MoS_2_ (Zhu et al., 2017). Ar-plasma treatment can effectively trigger the lateral sliding of the top S layer, thus obtaining 1T@2H-MoS_2_ mosaic structures with a 1T fraction of up to 40%. Although controllable and scalable, these two methods need further improvement because the 1T content in products is usually lower than 50%, which probably resulted from the limited sulfur vacancies formed in the lattice.

### HER performance of 1T-MoS_2_ catalysts

As a promising catalyst in the field of electrochemical hydrogen evolution, 1T-MoS_2_ has attracted much attention in recent years. Wang et al. prepared 1T/2H-MoS_2_ through NH_4_^+^-intercalation, which showed an excellent HER performance with a low overpotential of 234 mV at a current density of 10 mA/cm^2^ (η_10_) and a small Tafel slope of 46 mV dec^-1^ due to the enhanced conductivity and activated basal planes (Wang et al., 2017). As a comparison, the 2H-MoS_2_ had a much higher η_10_ of 309 mV and a larger Tafel slope of 89 mV dec^−1^. Lukowski et al. reported a greatly improved HER activity of metallic 1T-MoS_2_ nanosheets with a η_10_ of 187 mV and a Tafel slope of 43 mV dec^−1^ (2H-MoS_2_ with a η_10_ of 320 mV and a Tafel slope of 110 mV dec^−1^) prepared by chemical lithium intercalation, which was attributed to the fast electrode kinetics and proliferated density of catalytic active sites (Lukowski et al., 2013). Furthermore, porous MoS_2_ nanosheets with a dominant phase of 1T and a large number of edges and sulfur vacancies prepared by a facile LAAL strategy exhibited the best HER activity (a η_10_ of 153 mV and a Tafel slope of 43 mV dec^−1^) until now for the bare MoS_2_ catalysts (Yin et al., 2016).

Elemental doping is an effective route to regulate the d-band structure and hydrogen adsorption free energy (ΔG_H_), thus further improving the HER activity of 1T-MoS_2_. Recently, the inert basal plane of 2H-MoS_2_ was activated by atomic Pd doping through a spontaneous interfacial redox method. Structural characterization revealed that Pd substituted the Mo sites and produced sulfur vacancies at the same time, thus converting the partial 2H phase into the stabilized 1T phase (Luo et al., 2018). Theoretical calculation results indicated a ΔG_H_ of -0.02 eV at the sulfur sites of neighboring Pd atoms, which is highly active for the HER. Finally, an optimized Pd-MoS_2_ catalyst with 1.0 wt% Pd doping had a small η_10_ of 78 mV and an excellent stability after 5,000 cycles, which is much better than that of pristine 2H-MoS_2_ with a η_10_ of 328 mV and a Tafel slope of 157 mV dec^−1^. Qi et al. reported a single-atom Co doped distorted 1T-MoS_2_ nanosheet (SA Co-D 1T-MoS_2_) which demonstrated the lowest η_10_ of only 42 mV in all the reported MoS_2_ catalysts (Qi et al., 2019). The extraordinary HER activity was assigned to the ensemble effect of Co and S, which facilitated the hydrogen adsorption at the interface with ΔG_H_ of 0.03 eV. Atomic Cu-doped 1T-MoS_2_ (Cu@MoS_2_) also showed promising HER performance with a η_10_ of 131 mV and a small Tafel slope of 51 mV dec^−1^. Structural characterization and theoretical analysis demonstrated that single-atom Cu doping not only stabilized the 1T phase but also facilitated the charge transfer (Ji et al., 2019). Deng et al. synthesized a novel N-doped and PO_4_^3-^-intercalated 1T/2H-MoS_2_ array ((N, PO_4_^3-^)-MoS_2_/VG), which displayed a superior HER activity with a small η_10_ and Tafel slope of 85 mV and 42 mV dec^-1^, respectively (MoS_2_/VG with η_10_ of 187 mV and Tafel slope of 120 mV dec^-1^) (Deng et al., 2019). The outstanding HER performance was ascribed to the synergistic effect of N doping and PO_4_^3-^ intercalation, which decreased the band gap and lowered the d-band center and ΔG_H_.

Integrating 1T-MoS_2_ with conductive substrates such as carbon materials could not only stabilize the 1T phase but also increase the amount of exposed active sites to enhance the HER performance. For example, NH_4_^+^-intercalated 1T-MoS_2_ nanosheets grown on flexible single-walled carbon nanotubes (1T-MoS_2_/SWNT) exhibited a η_10_ as low as 108 mV and negligible activity loss after 3,000 cycles (Liu Q. et al., 2017). Electron donation from SWNT to 1T-MoS_2_ at the interface was beneficial for stabilizing the 1T phase and weakening the hydrogen adsorption energy. In addition, the ultra-small size of 1T-MoS_2_ nanopatches endowed a high density of active edges and basal planes. Wang et al. first constructed MoS_2_ nanoparticles on a three-dimensional carbon fiber paper to expose more edge sites and then conducted Li electrochemical intercalation to induce 1T phase formation and improve the electrical conductivity (Li-MoS_2_/CFP). Consequently, an ultrahigh HER activity with a η_10_ of 118 mV and a Tafel slope of 62 mV dec^−1^ was achieved (Wang H. et al., 2014). Similarly, reduced graphene oxide (RGO) was utilized as a template to grow 1T-MoS_2_ nanosheets, which donated electrons and promoted the 1T content from 15% to 50% (Cai et al., 2017). Accordingly, the 2H-MoS_2_ nanosheets exhibited a larger η_10_ of 348 mV and a Tafel slope of 90 mV dec^−1^. In contrast, the 1T-2H/RGO composites obtained a quite small η_10_ and a Tafel slope of 126 mV and 35 mV dec^−1^ due to numerous surface active sites and excellent charge transfer ability.

The aforementioned HER performance was obtained in the acidic medium (0.5 M H_2_SO_4_); however, developing efficient MoS_2_ HER catalysts under alkaline conditions will be more challenging because oxygen evolution reaction (OER) catalysts are usually unstable in the acidic medium. Gao et al. synthesized a highly efficient and stable carbon-doped 1T-2H/MoS_2_ nanosheets with 1T fraction of 60%, which exhibited a superb HER performance with a η_10_ of only 40 mV and Tafel slope of 46 mV dec^-1^ in 1.0 M KOH (Gao et al., 2020a). This is significantly reduced compared with those of 2H-MoS_2_/graphene oxide which has a η_10_ of 254 mV and a Tafel slope of 169 mV dec^−1^. The excellent electrochemical activity and stability was acquired by fast charge transfer and abundant active sites. The SA-Ru-MoS_2_ achieved a η_10_ as small as 76 mV in 1.0 M KOH (pure 2H-MoS_2_ with a poor η_10_ of 339 mV), which was attributed to reduced ΔG_H_, increased electrical conductivity, and modulated electronic structure (Zhang et al., 2019). Zhang et al. grew nickel hydr(oxy)oxide nanoparticles on the surface of 1T-MoS_2_ nanosheets to obtain 1T-MoS_2_/Ni^2+δ^O_δ_(OH)_2-δ_ hybrids, which displayed an excellent HER performance in 1.0 M KOH with a η_10_ of 73 mV and 185 mV smaller than those of the pristine 1T-MoS_2_. A mechanism study indicated that Ni^2+δ^O_δ_(OH)_2-δ_ nanoparticles promoted the adsorption and dissociation of H_2_O, hence providing sufficient H^+^ to produce H_2_ on the surface of 1T-MoS_2_ nanosheets (Zhang and Liang, 2018). Shang et al. embedded vertical monolayer 1T-MoS_2_ on the amorphous CoOOH substrate (MCSO), where the CoOOH substrate not only stabilized the metallic phase but also anchored the vertical 1T-MoS_2_ nanosheets to provide plenty of active sites (Shang et al., 2018). A small Tafel slope of 42 mV dec^−1^ and good stability of 25-h run time were achieved in the alkaline medium. Table 1 lists the HER performance of some representative 1T-MoS_2_ catalysts in both acidic and alkaline media in recent years. The phase modulation method, 1T content, and substrate were also summarized.

**TABLE 1:** HER performance of 1T-MoS_2_ catalysts in the reported literature.

Catalyst	Phase modulation method	1T content (%)	Substrate	Electrolyte	η_10_ (mV)	Tafel slope (mV dec^−1^)	Stability	Ref
1T/2H-MoS_2_	NH_4_^+^-intercalation	61.5	—	0.5 M H_2_SO_4_	234	46	1000 cycles	Wang et al. (2017)
1T-MoS_2_ nanosheets	Li^+^-intercalation	—	—	0.5 M H_2_SO_4_	187	43	9 h	Lukowski et al. (2013)
Porous 1T-MoS_2_	Li^+^-intercalation	82	—	0.5 M H_2_SO_4_	153	43	1000 cycles	Yin et al. (2016)
1T/2H-MoS_2_-HN	NH_4_^+^-intercalation	65	—	0.5 M H_2_SO_4_	156	47.9	1000 cycles	Wang et al. (2019)
1T-MoS_2_ quantum dots	Electrochemical Li^+^-intercalation	94	CFP^a^	0.5 M H_2_SO_4_	92	44	10000 cycles	Chen et al. (2018)
Ni-1T-MoS_2_	AA-intercalation	—	—	1.0 M KOH	199	52.7	—	Wang et al. (2022)
Pd-MoS_2_	Pd doping	—	—	0.5 M H_2_SO_4_	78	62	5,000 cycles	Luo et al. (2018)
SA Co-D 1T-MoS_2_	Co doping	—	—	0.5 M H_2_SO_4_	42	32	10000 cycles	Qi et al. (2019)
Cu@MoS_2_	Cu doping	—	—	0.5 M H_2_SO_4_	131	51	25000 s	Ji et al. (2019)
(N, PO_4_^3-^)-MoS_2_/VG	N doping plus PO_4_^3-^ intercalation	41	Graphene	0.5 M H_2_SO_4_	85	42	10 h	Deng et al. (2019)
Fe-MoS_2_ nanoflower	Fe doping	66	—	0.5 M H_2_SO_4_	136	82	1000 cycles	Zhao et al. (2017)
In-plane 1T/2H-MoS_2_	P doping	—	—	1.0 M KOH	320^b^	65	1000 cycles	Wang et al. (2018)
3D MoS_2_/candle soot/Ni foam	Ar bombardment	—	Ni foam	1.0 M KOH	56	49	46 h	Gao et al. (2020b)
1T-MoS_2_/SWNT	NH_4_^+^-intercalation	60	SWNT	0.5 M H_2_SO_4_	108	36	3,000 cycles	Liu et al. (2017a)
Li-MoS_2_/CFP	Electrochemical Li^+^-intercalation	—	CFP	0.5 M H_2_SO_4_	118	62	7000 cycles	Wang et al. (2014a)
1T-2H/RGO	NH_4_^+^-intercalation	50	RGO	0.5 M H_2_SO_4_	126	35	1000 cycles	Cai et al. (2017)
C + MoS_2_@GR-10W	Carbon doping	60	Graphene	1.0 M KOH	40	46	30 h	Gao et al. (2020a)
SA-Ru-MoS_2_	Ru doping	—	—	1.0 M KOH	76	21	—	Zhang et al. (2019)
1T-MoS_2_/Ni^2+δ^O_δ_(OH)_2-δ_	NH_4_^+^-intercalation	—	—	1.0 M KOH	73	75	5,000 cycles	Zhang and Liang, (2018)

a CFP: carbon fiber paper.

b η_20_: current density of 20 mA/cm^2^.

## Summary and perspectives

Herein, we first introduced a series of characterization techniques to confirm the presence and determine the content of 1T-MoS_2_. Then, some frequently used phase modulation strategies were summarized to realize the targeted synthesis and stabilization of 1T-MoS_2_. Finally, we presented some recent progress in improving the HER performance of 1T-MoS_2_ in both acidic and alkaline media including sulfur vacancy engineering, elemental doping, and integration with a conductive carbon substrate. However, there is still a possibility to optimize the synthesis and design efficient 1T-MoS_2_ HER catalysts for future research. 1) The synthetic parameters for controllable synthesis and stabilization of the metallic 1T phase. 2) Further modification or functionalization strategies for tuning the electronic structure and stabilizing the 1T phase need to be explored. 3) Further improvement in the HER performance of 1T-MoS_2_ in alkaline or neutral media due to the sluggish kinetics. 4) Investigating the synergism between 1T-MoS_2_ and other conductive substrates, such as nickel foam, which not only promote the exposure of more active sites but also offer better electrical conductivity.
